# Assessing the influence of rapid diagnostic test (RDT) accuracy on malaria misdiagnosis and antimalarial resistance in Nigeria

**DOI:** 10.1097/MS9.0000000000002925

**Published:** 2025-02-11

**Authors:** Promise Udohchukwu Okereke, Bamimore Mubarak Ayodeji, Roqeebat Titilope Bolarinwa, Oluwatobi Deborah Ayodeji, Idris Olayemi Popoola, Wisdom Obumneme Okereke, Nzubechukwu Ogamba, Lilian Uzoma Nworah, Chukwuemeka Victor Umeh, Chidubem Peter Okpechukwu, Solomon Nnayelugo Ibekwe, Emmanuel Emeka, Olalekan Simeon Tola

**Affiliations:** a Department of Dentistry and Dental Surgery, Faculty of Dentistry, College of Medicine, University of Nigeria, Enugu, Nigeria; b Dental Centre, University College Hospital, Ibadan, Nigeria; c Youth Health Action Network (YOHAN Research Institute), Enugu, Nigeria; d Department of Nursing, Ladoke Akintola University of Technology, Ogbomoso, Oyo State, Nigeria; e Department of Medicine and Surgery, Faculty of Medicine, College of Medicine, University of Nigeria, Enugu, Nigeria; f Irrua Specialist Hospital, Irrua, Nigeria; g University of Nigeria Teaching Hospital, Enugu, Nigeria; h Belgorod State National Research University, Belgorod, Russia

**Keywords:** antimalarial, malaria, misdiagnosis, rapid diagnostic testing, resistance

## Abstract

Malaria remains a critical health challenge in tropical regions, demanding accurate and timely diagnosis to prevent severe negative outcomes. The disease’s misdiagnosis, driven by inadequate diagnostic tools, insufficient training, and systemic health care deficiencies, complicates effective management. Rapid diagnostic tests (RDTs), commonly used in Africa, often produce false-negative results due to various factors like parasite density and test conditions, which can lead to inappropriate treatment and potential drug resistance. Microscopy, while the gold standard, is often unavailable in resource-limited settings, pushing reliance on less accurate methods. Technological advances and improved education for health care workers are important for diagnostic accuracy. Innovations such as the Nanomal DNA analyzer, a point-of-care device, offer quick, reliable testing and the potential to identify drug resistance markers. The accuracy of malaria treatment can be significantly improved by integrating clinical assessments with refined diagnostic methods, reducing the disease burden in endemic areas. This comprehensive approach, combining technology, systemic health care improvements, and policy alignment, is important for effective malaria management and eventual eradication in affected regions.

## Introduction

Malaria is a life-threatening disease primarily found in tropical countries (WHO, 2024)^[1]^. Recently, it was estimated that nearly 2000 African children die each day as a result of severe falciparum malaria^[2]^, a depressing figure that has changed little since 2015. This terrible death toll is a major justification for the substantial global investments in malaria control^[3]^ Supported by Watson *et al.*^[4]^, it was discovered that about 266 000 children in sub-Saharan Africa were killed by malaria in 2017^[4]^. Nigeria is one of the countries in the world with the highest burden of malaria, accounting for a quarter of all cases in Africa. According to the Centers for Disease Control and Prevention (CDC), microscopic examination remains the gold standard for laboratory confirmation of malaria. However, the policy and practice of presumptive treatment of malaria for all febrile illnesses has been widely advocated in sub-Saharan Africa. Pre-symptoms of malaria result in overdiagnosis and consequently overtreatment^[5]^. Despite its alarming incident and prevalent rate, it is still curable. However, the severity of the disease can progress to a fatal one if there is no effective, prompt, and accurate diagnosis (WHO, 2024)^[1]^. Early accurate diagnosis and risk assessment for malaria are crucial for improving patients’ terminal prognosis and preventing them from progressing to a severe or critical stage^[6]^.

## Diagnosis and misdiagnosis

A major playmaker in the goal of malaria elimination is the issue of proper diagnosis^[7]^. Therefore, proper diagnosis is expedient for the adequate management of malaria, thereby shortening the time of recovery and ensuring the possibility of drug resistance is minimized^[7]^. Till the present time, various studies highlighted different factors associated with the misdiagnosis of malaria such as insufficient diagnostic equipment, means of diagnosis, lack of clinical supervision/training for local clinicians, lack of malaria-related knowledge, and health system factors^[6]^.

Rapid diagnostic test (RDT), microscopy, polymerase chain reaction (PCR), and loop-mediated isothermal amplification (LAMP) are some of the most adopted methods that can be used for malaria diagnosis with each of the methods having their strength(s) and limitation(s) based on accuracy, cost availability of trained personnel, and infrastructure [7,8]. Of all, microscopy is regarded as the gold standard method for malaria diagnosis (WHO, 2024; CDC, 2023)^[1,9-12]^, although RDT is the primary mode of malaria diagnosis in Africa, accounting for 75% of diagnostic testing for suspected malaria in public facilities in 2017^[4]^. RDT works by detecting the causative parasite of malaria at densities associated with the disease, helps to distinguish malaria from other types of diseases, and is widely adopted in situations where microscopy-based diagnosis is not available (WHO, 2024)^[1]^.

Despite the ease portrayed by RDT, the density of the parasite detected can also affect the accuracy through sensitivity (Yigezu *et al.*, 2024)^[8]^. Sensitivity is determined by the quality of manufacture, species, number, viability and strain of parasites present, condition of the RDT (including storage conditions), technique and care used in performing the test, and reader’s interpretation (WHO, 2024)^[1]^. However, false-negative RDT results are known to occur for a variety of reasons, including operator error, poor storage conditions, *Plasmodium falciparum* histidine-rich protein 2/3 (pfhrp2/3) gene deletions, poor performance of specific RDT brands, and low-parasite density infections^[4]^. This type of error can lead to the prescription of an ineffective drug course, to which the parasite is likely to be resistant, or that does not provide the required protection against relapse^[11]^. However, it has been shown that RDT has more accuracy and sensitivity than microscopy with *var*ATS qPCR as reference although both seem to have similar specificity^[13]^.

Apart from RDT, overreliance on clinical diagnosis without laboratory diagnosis is also an important factor in the misdiagnosis of malaria. The clinical diagnosis by health practitioners, which is widely used, is notably inexpensive and is prone to errors due to other febrile conditions that manifest similar symptoms compared to malaria^[7]^. This was also supported by previously published literature, which stated that sporadic and inadequate availability of RDT and microscopy, reliance on strong clinical suspicion of malaria, mistrust in the performance of microscopy and RDT tests in diagnosing malaria, and a lack of alternative diagnosis are the causes of overreliance on clinical diagnosis without parasitological evidence^[10]^. The dependence on the clinical diagnosis without having a corroboration with the laboratory diagnosis has been unreliable (sensitivity of 47.2%) with possible outcomes of misdiagnosis, treatment failure, and emergence of drug resistance^[7]^.

As a solution, improving health care workers training and implementing better diagnostic methods are crucial for effective malaria control^[8]^. Also, there can be reduction in the misdiagnosis of malaria by combining clinical and diagnostic methods^[11]^.

## The impact of misdiagnosis of malaria on the reported prevalence rate

The estimated global incident cases of malaria in 2022 was 249 million with 608 000 malaria deaths in 85 countries. Four African countries carried the highest burden with Nigeria alone accounting for approximately 26.8% of global malaria fatalities.

However, the impact of the misdiagnosis of malaria on these statistics cannot be overlooked as the majority of “malaria” cases, i.e., febrility (fever >38 °C), are self-diagnosed and most treatments and deaths occur at home. A study was conducted on 1193 febrile patients between 2008 and 2010 in Cambodia by the World Health Organization (WHO). Of the 1193 patients, only about 47% had malaria (*P. falciparum* and *Plasmodium vivax*)^[14]^. A similar study was carried out on 870 febrile patients in Tanzania in 2013. Of the 870 patients, 528 patients were admitted with a diagnosis of malaria. However, only 14 (1.6%) had malaria as the actual cause of their fever^[14]^. Another study was carried out on 341 children aged ≤5 years at Kenyatta National Hospital in Nairobi, Kenya. This study revealed that in 44 (12.9%) of the children, prescription of antimalarial drugs despite negative microscopy results was found with mortality reported in 48 (14.1%)^[15]^. These have shown that the misdiagnosis of malaria has significantly increased morbidity and mortality rates and should be strictly regulated.

## Role of technology and education in reducing misdiagnosis

In the successful control and elimination programs of malaria, malaria diagnostics tests and methods have been reviewed as being key factors^[16]^. As the drive toward the elimination of malaria intensifies, the search for ideal, simple, fast, and reliable point-of-care (POC) diagnostic tools is needed more than ever before, to be used in conjunction with a functional surveillance system supported by the ideal vaccine^[7]^. In a bid, several technological innovations that have contributed significantly to malaria response have been developed across the world^[16]^.

According to the CDC in 2020, from the pathological organism point of view, various biological-based diagnostic methods have been developed over the years, ranging from the popular Giemsa-violet microscopic method to molecular-based diagnostics (CDC, 2020)^[9]^. More recent developments in malaria diagnostics have focused on the detection and amplification of *Plasmodium* nucleic acids. Like antigen detection, which allows the detection of *Plasmodium* spp. directly, these methods are often far more sensitive and able to detect parasites at a much lower density because of the nucleic acid amplification from a small amount of DNA^[11]^. Furthermore, many new molecular techniques such as the PCR, LAMP, and sequencing methods allow more data to be gathered about the identification of *Plasmodium* spp. and improve the characterization of the DNA sequences and loci of interest^[11]^.

Several devices have also been developed to improve malaria diagnosis, and these include the nanomal DNA analyzer, a simple, rapid and affordable POC handheld diagnostic nanotechnology device to confirm malaria diagnosis and detect drug resistance in malaria parasites in minutes and at the patient’s side. It does this by analysis of mutations in malaria DNA using a range of proven nanotechnologies^[16]^. To help guide research and development efforts in this area, WHO has developed two preferred product characteristics (PPCs) to detect the risk of *P. vivax* relapse. The first PPC is for POC tests to identify individuals at risk of relapse to guide radical cure and case management; individuals at risk of *P. vivax* relapse include those living in endemic areas, migrants, and those inadequately treated, particularly without anti-relapse therapy like primaquine or tafenoquine. Immunocompromised individuals, children, and pregnant women also face elevated risk^[17]^. The second PPC is for laboratory-based tests to screen communities or individuals, facilitating surveillance and monitoring efforts for *P. vivax* control and elimination (WHO, 2024)^[1]^. These tools aim to improve screening, the use of radical cure and case management among high-risk populations, and support population-level risk stratification for the targeting of interventions and the monitoring and evaluation of ongoing elimination programs (WHO, 2024)^[1]^.

As a comparative form of analysis, the PCR-based methods are more effective than microscopy and are increasingly utilized in larger laboratories that can receive blood samples from smaller facilities. The LAMP has similar performance rates to PCR for diagnosis of *P. falciparum* and is perhaps more practical in low-resource endemic settings (Tambo *et al.*, 2018)^[11,18]^. By using the power of the recent methods, we can provide a broader understanding of the multiplication of infections and or transmission dynamics of *Plasmodium* spp. This will result in improved disease control strategies, better-informed policy, and effective treatment for malaria-positive patients^[11]^. In addition, biosensors outperform traditional laboratory procedures in terms of analytical performance^[12]^. This is due to its simplicity of application, little information requirement, the capability of detecting >100 parasites/μl, and quick results with a turnaround time of less than 30 mins^[7]^. Improved diagnostic technologies that can detect extremely low-parasite concentrations are needed to allow for targeted treatment of affected people^[12]^.

## Anti-malaria resistance

It is not news that malaria is a great threat to Nigeria’s health system. While there are several medications in place to combat the malaria parasites, the growing resistance of malaria parasites to antimalarial medication has made the threat seem invincible.

Five species of *Plasmodium* affect humans and cause illnesses. They are *P. falciparum, Plasmodium malariae, P. vivax, Plasmodium ovale*, and *Plasmodium knowlesi*. Of all these species, *P. falciparum* causes the most lethal malaria in African countries.^[17]^ One of the oldest primary chemotherapeutic means used to treat malaria is chloroquine, as it is cheap and effective. However, in 1957, the first chloroquine-resistant strain of *P. falciparum* was observed in South East Asia.^[19]^ Few decades later, the chloroquine-resistant strain had spread through all the countries in tropical Africa. Currently, Artemisinin-based Combination Therapy has been widely adopted in place of chloroquine as the most effective treatment for *P. falciparum* malaria^[20]^.

A critical factor to be considered in the management of malaria is an effective and accurate diagnosis. Most commonly in rural areas, malaria tends to be treated symptomatically and not backed up with accurate diagnostic tests. So, various illnesses that have similar presenting symptoms with malaria tend to be misdiagnosed. Some of these are dengue fever, typhoid fever, pneumonia, and even common cold. When patients present with feverish symptoms, health care providers often prioritize malaria diagnosis first, followed by typhoid fever as a secondary consideration, which may even be treated empirically without definitive testing (Lawrence *et al.*, 2021)^[21]^.

In malaria-endemic regions in Africa, particularly in areas with limited access to health care services, the easy accessibility of antimalarial medications has been abused, as self-diagnosis and improper regulation of medications are prevalent. Not only will this lead to worse conditions, but it has also been found to contribute to drug resistance. In addition, this has contributed immensely to the rise in children’s morbidity rate as misdiagnosis consequently leads to missed opportunities to treat real illnesses. Consequently, WHO recommended that all suspected cases be subjected to parasite-based diagnostic testing (either through microscopy or RDT)^[2]^.

RDT emerged and was adopted in the 1990s, to counteract the misdiagnosis and co-infection of malaria with other infections that have similar clinical presentation. RDT outshone the existing microscopic laboratory testing as it offered accurate diagnosis and required less technical skill.^[22]^

The WHO considers microscopic examination to be the most reliable and definitive method for laboratory confirmation of malaria, setting the benchmark for diagnostic accuracy.^[2]^ However, the policy of the presumptive treatment of malaria has been widely adopted in several regions in Africa.^[23]^

## Recommendations

The unavailability of easy-to-use and accurate diagnostic tools coupled with the increasing morbidity potentials of malaria justified the opportunistic presumptive treatment. Hence, for several years, malaria was considered the primary cause of fever especially if accompanied by headache and vomiting^[24]^.

The forefront recommendation to eliminate malaria in Nigeria would be the total elimination of the Presumptive Treatment of Malaria policy. Antimalarials should only be prescribed based on confirmed laboratory testing (CDC, 2023)^[9]^ In 1945, Alexander Fleming stated in his Nobel Lecture that “the ignorant man may easily underdose himself, and by exposing his microbes to non-lethal quantities of the drug, he makes them resistant.” (Michal, 2020)^[25]^.

Therefore, to further improve malaria prevention and reduce misdiagnosis, the following recommendations can help if adequately implemented accordingly.

1. **Improve RDT quality control and standardization**

There should be rigorous quality control measures put in place by relevant stakeholders to ensure that the RDTs used in Nigeria consistently meet international diagnostic standards. The government, in collaboration with regulatory bodies such as the Nigerian Centre for Disease Control, should regularly monitor and assess the accuracy of the RDTs across different regions to prevent discrepancies in diagnosis.

2. **Training and capacity building for health workers**

There is need for continuous training of health workers on the correct use and interpretation of RDTs through Continuing Professional Development programs and workshops. Also, proper diagnostic procedures should be emphasized to avoid false positives or negatives that could contribute to misdiagnosis. Refresher training sessions and the inclusion of diagnostic accuracy assessments in performance evaluations of relevant health personnel could help reduce errors.

3. **Integration of RDT use with microscopy in high-burden areas**

The combination of RDTs with microscopy in areas with high malaria prevalence would enhance diagnostic accuracy. While RDTs provide quick results, microscopy remains the gold standard for malaria diagnosis. In cases of conflicting results, especially with suspected drug resistance or complicated malaria, microscopy should always serve as a confirmatory test.

4. **Public awareness campaigns on RDTs and malaria diagnosis**

Public health campaigns should be aimed and tailored to educate the population on the importance of accurate diagnosis before the use of antimalarial drugs. These campaigns should also address the dangers of self-medication, the overreliance on presumptive treatment, and the need to trust health workers’ diagnostic efforts. This could curb unnecessary antimalarial use, which is a key factor in the rise of drug resistance.

5. **Large-scale studies on RDT performance across regions**

A large-scale, region-specific study on the performance of RDTs in different regions or geopolitical zones of Nigeria would be crucial. The study should assess different parameters such as sensitivity and specificity, which might affect the accuracy of RDTs. The data collected can guide better procurement policies, ensuring that only the most reliable RDTs are distributed in the country.

6. **Encourage rational antimalarial drug prescription**

It is vital to strengthen the enforcement of guidelines on rational antimalarial prescription. Health professionals should strictly adhere to treatment protocols based on confirmed diagnosis, whether through RDT or microscopy, to prevent the misuse of antimalarial drugs and the consequent development of resistance.

7. **Expand research on antimalarial drug resistance patterns**

Continuous research on the genetic patterns of *Plasmodium* species and their evolving resistance to antimalarials should be encouraged. Surveillance systems, particularly in regions with known resistance, should be established to monitor emerging resistance trends and guide public health interventions.

8. **Incorporate technological advancements in diagnostics**

Innovations in diagnostic tools, such as molecular tests that complement RDTs, should be explored. These new technologies, though more expensive, could help detect low-level parasitemia and mixed infections, which RDTs may miss, thereby improving diagnostic precision and reducing the risk of misdiagnosis.

9. **Enhance collaboration with global health partners**

Nigeria should improve its collaboration with global health organizations such as the World Health Organization (WHO) and other relevant organizations to access resources, technical assistance, and the latest diagnostic technologies in combating the deleterious effects of malaria infections. This will aid in combating malaria misdiagnosis and controlling the spread of antimalarial resistance.

10. **Strengthen health information systems**

There is a need for better tracking and reporting of RDT performance, malaria cases, and antimalarial drug usage. Strengthening health information systems to monitor trends in diagnostic accuracy and resistance patterns would help policymakers and stakeholders make data-driven decisions for controlling malaria and preventing resistance.

By implementing these recommendations, Nigeria can significantly reduce malaria misdiagnosis and limit the spread of antimalarial resistance, ensuring that malaria control efforts are more effective and sustainable in the long term.

## Conclusion

The responsible use of antimalarial medications as well as countering misdiagnosis is a collective obligation that calls for the collaboration of health care workers as well as patients. Addressing the influence of RDT accuracy on malaria misdiagnosis and antimalarial resistance in Nigeria is critical to improve the country’s malaria control efforts. Ensuring the reliability of RDTs through quality control, training health workers, and integrating diagnostic methods can significantly reduce misdiagnoses. Furthermore, public education on accurate diagnosis and the rational use of antimalarial drugs is essential to curbing the misuse of treatments, which contributes to drug resistance. By promoting continuous research, technological advancements, and global collaboration, Nigeria can effectively combat the dual challenges of misdiagnosis and antimalarial resistance. These combined efforts will strengthen the health system’s response to malaria, ultimately leading to better patient outcomes and a more sustainable fight against this endemic disease.

## Data Availability

Not applicable.
